# Smad3 Promotes Cancer‐Associated Fibroblasts Generation via Macrophage–Myofibroblast Transition

**DOI:** 10.1002/advs.202101235

**Published:** 2021-11-17

**Authors:** Philip Chiu‐Tsun Tang, Jeff Yat‐Fai Chung, Vivian Wei‐wen Xue, Jun Xiao, Xiao‐Ming Meng, Xiao‐Ru Huang, Shuang Zhou, Alex Siu‐Wing Chan, Anna Chi‐Man Tsang, Alfred Sze‐Lok Cheng, Tin‐Lap Lee, Kam‐Tong Leung, Eric W.‐F. Lam, Ka‐Fai To, Patrick Ming‐Kuen Tang, Hui‐Yao Lan

**Affiliations:** ^1^ Department of Anatomical and Cellular Pathology State Key Laboratory of Translational Oncology The Chinese University of Hong Kong Hong Kong China; ^2^ Department of Medicine and Therapeutics Li Ka Shing Institute of Health Sciences The Chinese University of Hong Kong Hong Kong China; ^3^ School of Pharmacy Anhui Medical University An Hui China; ^4^ Guangdong‐Hong Kong Joint Laboratory on Immunological and Genetic Kidney Diseases Guangdong Academy of Medical Sciences Guangdong Provincial People's Hospital Guangzhou China; ^5^ Department of Histology and Embryology Tongji University School of Medicine Tongji University Cancer Institute Shanghai China; ^6^ Department of Applied Social Sciences The Hong Kong Polytechnic University Kowloon Hong Kong China; ^7^ School of Biomedical Sciences The Chinese University of Hong Kong Hong Kong China; ^8^ Reproduction Development and Endocrinology Program School of Biomedical Sciences The Chinese University of Hong Kong Hong Kong China; ^9^ Department of Paediatrics Prince of Wales Hospital The Chinese University of Hong Kong Hong Kong China; ^10^ Sun Yat‐sen University Cancer Center State Key Laboratory of Oncology in South China Collaborative Innovation Center for Cancer Medicine, Sun Yat‐sen University Guangzhou Guangdong 510060 China; ^11^ The Chinese University of Hong Kong‐Guangdong Academy of Sciences/Guangdong Provincial People's Hospital Joint Research Laboratory on Immunological and Genetic Kidney Diseases The Chinese University of Hong Kong Hong Kong China

**Keywords:** cancer‐associated fibroblasts, macrophage–myofibroblast transition, Smad3, tumor‐associated macrophages, tumor microenvironment

## Abstract

Cancer‐associated fibroblasts (CAFs) are important in tumor microenvironment (TME) driven cancer progression. However, CAFs are heterogeneous and still largely underdefined, better understanding their origins will identify new therapeutic strategies for cancer. Here, the authors discovered a new role of macrophage‐myofibroblast transition (MMT) in cancer for de novo generating protumoral CAFs by resolving the transcriptome dynamics of tumor‐associated macrophages (TAM) with single‐cell resolution. MMT cells (MMTs) are observed in non‐small‐cell lung carcinoma (NSCLC) associated with CAF abundance and patient mortality. By fate‐mapping study, RNA velocity, and pseudotime analysis, existence of novel macrophage‐lineage‐derived CAF subset in the TME of Lewis lung carcinoma (LLC) model is confirmed, which is directly transited via MMT from M2‐TAM in vivo and bone‐marrow‐derived macrophages (BMDM) in vitro. Adoptive transfer of BMDM‐derived MMTs markedly promote CAF formation in LLC‐bearing mice. Mechanistically, a Smad3‐centric regulatory network is upregulated in the MMTs of NSCLC, where chromatin immunoprecipitation sequencing(ChIP‐seq) detects a significant enrichment of Smad3 binding on fibroblast differentiation genes in the macrophage‐lineage cells in LLC‐tumor. More importantly, macrophage‐specific deletion and pharmaceutical inhibition of Smad3 effectively block MMT, therefore, suppressing the CAF formation and cancer progression in vivo. Thus, MMT may represent a novel therapeutic target of CAF for cancer immunotherapy.

## Graphical abstract

Here, the authors discovered a direct mechanism of tumor‐associated macrophages for de novo generating protumoral cancer‐associated fibroblasts (CAF) via macrophage‐myofibroblast transition (MMT) by resolving their transcriptome dynamics at single‐cell resolution. Encouragingly, targeting the MMT key regulator Smad3 effectively blocked the production and protumoral actions of CAF, evidencing its translational potential as a novel therapeutic target in the tumour microenvironment for cancer.

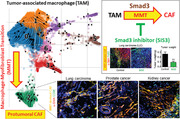

## 1 Introduction

Cancer‐associated fibroblasts (CAFs) are the most prominent stromal components;^[^ ^1^ ^]^ they are a type of myofibroblasts that notably enhance the malignancy and progression of cancer.^[^ ^2^ ^]^ Cancer cells are heterogeneous, versatile, and adaptable, leading to primary and secondary drug resistance.^[^ ^3^ ^]^ Therapies that target the tumor microenvironment (TME) show promise, as cancer growth, invasion, and metastasis rely on stromal conditions.^[^ ^4^ ^]^ The origins of CAFs are highly heterogeneous and still controversial.^[^ ^5^ ^]^ We recently discovered a Smad3‐dependent tumor microenvironment (Smad3‐TME) essential for promoting cancer progression,^[^ ^6^ ^]^ but its pathogenic mechanisms are still largely unknown especially on CAFs. A better understanding of the dynamics of Smad3‐TME may uncover novel therapeutic targets for blocking CAF formation.

Single‐cell RNA sequencing (scRNA‐seq) is an emerging approach for resolving cell‐to‐cell transcriptome profiles on a genomic scale. It has led to many profound discoveries in biology, which largely enhance the identification of novel cell types and therapeutic targets.^[^ ^7^ ^]^ Recently, we identified macrophage–myofibroblast transition (MMT) as a novel mechanism for the generation of pathogenic fibroblasts in the diseased kidney for tissue scarring.^[^ ^8^, ^9^, ^10^, ^11^ ^]^ Surprisingly, its potential implication in cancer is still unexplored. Indeed, tumor‐associated macrophages (TAM) are significantly correlated with CAF levels in lung carcinoma and neuroblastoma.^[^ ^12^, ^13^ ^]^ Moreover, macrophage marker expressing CAFs are suggested to be a poor prognostic indicator for oral squamous cell carcinoma.^[^ ^14^, ^15^, ^16^, ^17^ ^]^ However, the underlying mechanism of how macrophages promote CAFs is still largely unclear. Transcriptome signatures and phenotypes are highly dynamic in the TME, where TAM were transcriptionally distinct from monocytes and tissue‐resident macrophages due to the cancer cells.^[^ ^18^, ^19^ ^]^ The scRNA‐seq helps to dissect reconstituted temporal transcription networks in response to developmental processes and/or external stimuli, which can be masked at the population level.^[^ ^20^, ^21^, ^22^ ^]^ Elucidating the Smad3‐TME at the single‐cell resolution can be a promising research strategy for identifying how TAM promote CAF formation in cancer.

Here, we identified MMT as a new and direct mechanism for TAM to promote CAF formation. By 10x scRNA‐seq, we uncovered the involvement of MMT in cancer and their association with CAF formation and cancer mortality, especially in the non‐small‐cell lung carcinoma (NSCLC). We evidenced a new macrophage‐lineage CAF subpopulation in both experimental and human TMEs, which was derived from M2 TAM via MMT. Mechanistically, a unique Smad3‐centric gene network was reconstructed and detected as the key regulatory mechanism of MMT in NSCLC. More importantly, we demonstrated that macrophage‐specific silencing and pharmacological inhibition of Smad3 effectively blocked MMT, therefore, largely suppressing CAF formation and cancer progression in vivo. Thus, MMT may represent a novel therapeutic target of CAFs for anticancer immunotherapy.

## 2 Results

### 2.1 MMT Cells Show CAF Signatures in NSCLC

**Figure 1:**
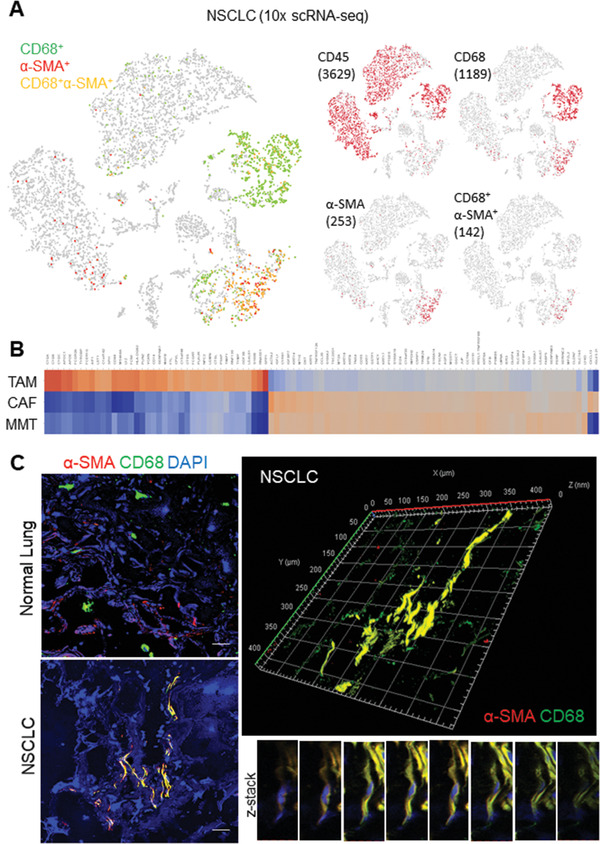
MMTs resemble as CAFs in NSCLC. A) 10x scRNA‐seq detects 142 MMTs (CD68^+^ *α*‐SMA^+^ cells) accounted for more than half of the CAF population (253 *α*‐SMA^+^ cells). B) Heat map showing the CAF‐like transcriptome of MMTs in the comparison of significant (*p* < 0.05) differentially expressed genes between TAM (CD68^+^ CD45^+^), CAFs (*α*‐SMA^+^), and MMTs (*α*‐SMA^+^CD68^+^) of NSCLC. C) Confocal imaging detected MMTs in NSCLC but not in normal lung tissue, and further demonstrated spindle‐like myofibroblast morphology of MMT cell by *z*‐stack scanning and reconstructed 3D modeling. Scale bar, 50 µm.

As the role of MMT in cancer is still unknown, we examined their characteristics at the transcriptomic level with 10x scRNA‐seq. Consistent to our notion, we detected MMT cells (MMTs; 142 *α*‐SMA^+^ CD68^+^ cells) in an NSCLC dataset, which contributed to more than half of CAF population (253 *α*‐SMA^+^ cells) (**Figure** **1**A). Interestingly, their transcriptional profiles were highly homologous to CAFs (*α*‐SMA^+^ CD68^−^) instead of TAM (*α*‐SMA^−^ CD68^+^) (Figure 1B). In addition, through 3D confocal imaging, we found MMTs showing a spindle‐like myofibroblast morphology in NSCLC, but absent in the normal lung tissue (Figure 1C). Encouragingly, we detected MMTs in a number of human TMEs as well as patient‐derived NSCLC xenografts A549 (Figure S1, Supporting Information), revealing a potential contribution of MMT in CAF development.

### 2.2 MMT‐Derived CAF Subset Originates from M2 TAM

**Figure 2:**
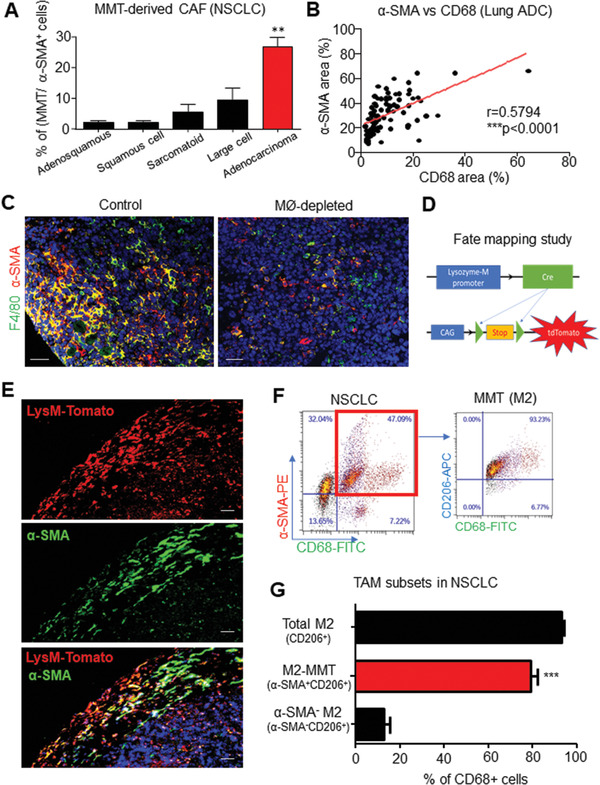
MMTs originate from protumoral M2 TAM. MMT highly occurred in A) adenocarcinoma of NSCLC (*p* < 0.01 adenocarcinoma compared with other NSCLC subtypes, one‐way analysis of variance (ANOVA), *n* = 121), B) where TAM (CD68) and CAF (*α*‐SMA) levels are positively correlated (Spearman correlation, *p* < 0.0001, *n* = 87) in our lung adenocarcinoma (ADC) cohort. C) Immunostaining showing diphtheria toxin (DT)‐driven macrophage ablation largely suppresses MMT‐dependent CAF formation (*α*‐SMA^+^CD68^+^) in LLC tumor on LysM‐Cre/ROSA‐DTR mice. D) Lineage tracing strategy of LysM‐Cre/ROSA‐tdTomato mice, where tdTomato open reading frame (ORF) is linked to a constant CAG promoter, which can be activated by macrophage‐specific Cre‐mediated stop codon excision, resulting in the continuous tdTomato expression in the macrophage lineage. E) Immunofluorescence visualizes macrophage‐lineage‐derived CAFs (*α*‐SMA^+^ tdTomato^+^) in LLC tumor at day 12. M2‐TAM are the major source of F) M2‐MMTs (α‐SMA+CD206+CD68+) in human NSCLC TME quantifying by G) flow cytometry analysis (***p < 0.001 M2‐MMT versus *α*‐SMA^−^ M2, one‐way ANOVA, *n* = 6). Scale bar, C) 50 and E) 25 µm.

Our in‐house cohort revealed that MMT occurred frequently in lung adenocarcinoma (ADC) compared to other subtypes of NSCLC (**Figure** **2**A), where TAM and CAF abundances were significantly correlated in lung adenocarcinoma (*n* = 87, *p* < 0.0001) (Figure 2B). As MMT may serve as a direct mechanism of TAM for promoting CAF development, we further evidenced the existence of macrophage‐lineage CAF in vivo. First, to elucidate the role of macrophages in CAF generation, we genetically depleted macrophages in our well‐established LysM‐Cre/diphtheria toxin (DT) receptor (LysM‐DTR) mice stain^[^ ^9^, ^11^, ^23^, ^24^ ^]^ as well as clodronate liposome‐driven macrophage depletion.^[^ ^25^ ^]^ Interestingly, macrophage depletion largely reduced MMT and CAFs in the Lewis lung carcinoma (LLC) tumors (Figure 2C; Figure S2, Supporting Information), implying a direct mechanism of TAM in CAF formation. Thus, we conducted a fate‐mapping study with our LysM‐Cre/ROSA26‐tdTomato transgenic mice,^[^ ^9^, ^24^ ^]^ where the macrophage‐lineage‐derived cells can be traced by their permanent tdTomato expression due to LysM‐driven Cre recombinase transcription (Figure 2D). In line with our hypothesis, we detected *α*‐SMA^+^ tdTomato cells in the LLC tumors (Figure 2E), which progressively contributed to the CAF formation, accounting for more than 40% of the total *α*‐SMA^+^ cells at the late phase of tumorigenesis since day 20 (Figure S3, Supporting Information), representing a new and rich CAF source. In concordance, the observation of MMT‐derived CAFs was confirmed by adoptive transferring of green fluorescent protein (GFP) expressing or Dil tracker dye labeled bone‐marrow‐derived macrophages (BMDM) in a LysM‐independent manner (Figure S4, Supporting Information).

Interestingly, we observed that MMTs were predominately expressing M2 marker CD206 in NSCLC, accounting for ≈80% of total CD68^+^ TAM in the specimens (Figure 2F,G). To provide evidence that the MMTs are derived from M2 cells, we cultured GFP‐expressing BMDM with interleukin 4 (IL‐4) to generate M2 macrophages as previously described^[^ ^26^ ^]^ and then adoptively transferred these M2 cells into LLC‐bearing mice. As shown in Figure S5 (Supporting Information), we detected M2‐derived CAFs in the LLC tumors indicating by their GFP expression, demonstrating M2 as a precursor of MMTs and MMT‐derived CAFs from the M2 TAM.

### 2.3 TAM Directly Transit into CAFs at Single‐Cell Resolution

**Figure 3:**
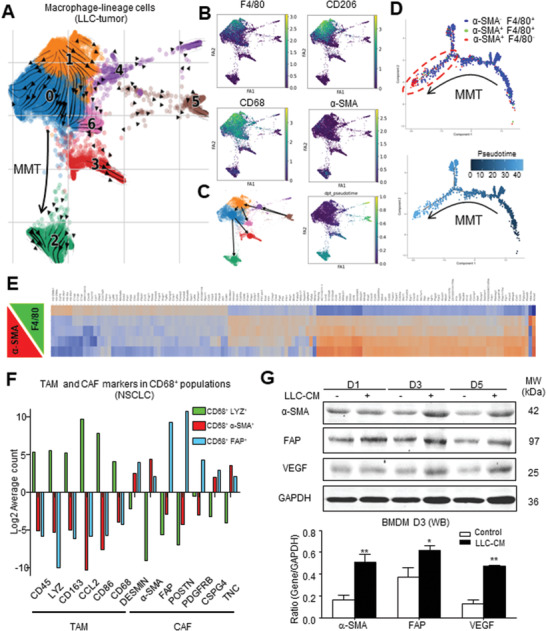
Macrophage‐lineage scRNA‐seq captures the de novo formation of CAFs from TAM. RNA velocity and pseudotime analysis showing the de novo generation of CAFs via MMT in the developmental trajectory of macrophage lineage since TAM (*α*‐SMA^−^F4/80^+^, cluster 1 in A‐C) RNA velocity projected plot, D) blue dots in pseudo‐timeline), to MMTs (*α*‐SMA^+^F4/80^+^, cluster 0, green dots), then finally to CAFs (*α*‐SMA^+^F4/80^−^, cluster 2, red dots), associated with the successive transcriptional change of a panel of TAM to CAF markers observed in E) murine macrophage‐lineage tracing study and F) human NSCLC. G) LLC‐derived cancer secretome (LLC‐CM) effectively induces macrophage–myofibroblast transition on BMDM in vitro, showing by the progressive expression of CAF markers (*α*‐SMA, FAP) and effector (VEGF) compared to the control in western blot (*p < 0.05 vs control, **p < 0.01 vs control, one‐way ANOVA, *n* = 3).

To show the contribution of MMT in CAF formation at genomic level, we flow‐sorted macrophage‐lineage cells from the LLC tumors of LysM‐Cre/ROSA26‐tdTomato transgenic mice for 10x scRNA‐seq analysis. We defined maturation stages of the macrophage‐lineage cells in an unsupervised manner, where cell–cell transcriptional similarity is represented by their distance in the t‐distributed stochastic neighbor embedding (t‐SNE) plot. Interestingly, the macrophage‐lineage CAFs (*α*‐SMA^+^ F4/80^−^) and TAM (*α*‐SMA^−^ F4/80^+^) formed two clusters, where MMTs (*α*‐SMA^+^ F4/80^+^) enriched in the adjacent region, implying an intermediate cell state (Figure S6A, Supporting Information). To resolve their developmental stages, we conducted trajectory inference based on RNA velocity of LysM‐tdTomato cells from LLC tumor, where unspliced and spliced messenger RNA (mRNA) ratio (i.e., RNA velocity) of individual cells was compared to accurately predict their cellular state during differentiation.^[^ ^27^ ^]^ As shown in **Figure** **3**A, we successfully recapitulated MMT in the RNA velocity projected plot, where differentiation of the macrophage‐lineage cells in the LLC tumor was started from cluster 1 with predominant F4/80^+^ cells into cluster 0 with F4/80^+^ CD206^+^ M2 and *α*‐SMA^+^ cells, then terminated at distinct cluster 2 with *α*‐SMA^+^ cells (Figure 3B,C; Figure S6C, Supporting Information).

We further ordered the macrophage‐lineage cells along a putative developmental trajectory from least (*α*‐SMA^−^ F4/80^+^) to most differentiated (*α*‐SMA^+^ F4/80^−^) (Figure S6A, Supporting Information); therefore, a pseudo‐timeline with bifurcation containing both MMTs and CAFs at the most differentiated timeline was obtained (Figure 3D). In line with this notion, we detected a successive transcriptional change (Figure 3E) and a diminishing number of macrophage marker expressions (Figures S3 and S6B, Supporting Information) in macrophage‐lineage cells that were strongly associated with an increment of *α*‐SMA in LLC tumors. Furthermore, we also detected a progressive increment of CAF markers but diminishing macrophage‐linage markers associated with the *α*‐SMA and fibroblast activation protein alpha (FAP) expressions in TAM of NSCLC (Figure 3F). Importantly, by in vitro assay, we confirmed that stimulation with the LLC cancer cells conditioned medium (LLC‐CM) was capable of inducing de novo expression of CAF antigens (*α*‐SMA, FAP) and effector (vascular endothelial growth factor (VEGF)) in BMDM but not the fibroblasts (Figure 3G; Figure S7, Supporting Information); clearly demonstrating a direct role of MMT in CAF formation. These findings evidenced MMT as a novel mechanism for generating CAFs directly from a rich source TAM.

### 2.4 MMT‐Derived CAFs Promote Cancer Progression

**Figure 4:**
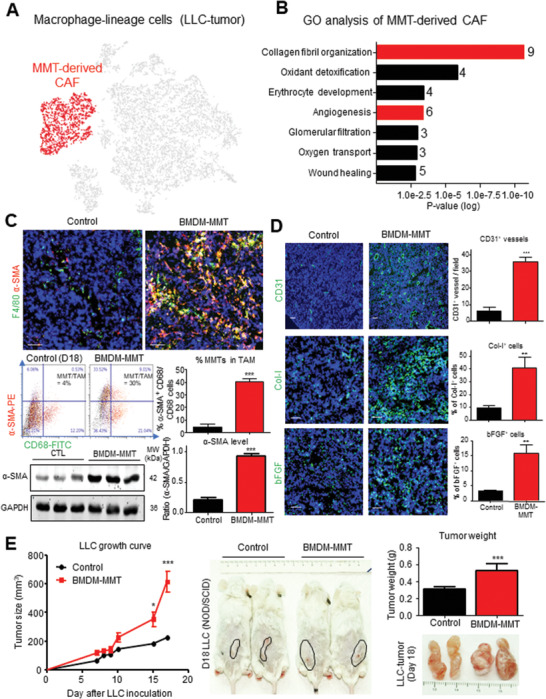
MMT generates angiogenic CAFs for tumor promotion. GO analysis (DAVID) reveals upregulated DEGs of A) macrophage‐derived CAFs (*α*‐SMA^+^ cells, red‐highlighted) in macrophage‐lineage scRNA‐seq of LLC‐TME were highly associated with B) protumoral CAF functions (collagen fibril organization, and angiogenesis). BMDM‐derived MMTs (BMDM‐MMT) adoptive transfer significantly increased the C) transition and CAF formation (% of MMTs in TAM population and *α*‐SMA protein level; *n* = 4, ***p < 0.01 vs control, *t*‐test) and D) angiogenesis (CD31, Col‐I, and bFGF; *n* = 4, ***p < 0.001, **p < 0.01 vs control, *t*‐test) detected by immunofluorescence, flow cytometry and western blotting and E) resulted in a dramatic acceleration of tumor growth in macrophage‐malfunctioned NOD/SCID mice in vivo (*p < 0.05, ***p < 0.001 versus control, one‐way ANOVA (growth curve), *t*‐test (tumor weight), *n* = 4). Scale bar, 50 µm.

We next examined the functional role of MMTs in cancer. Interestingly, gene ontology analysis revealed that the upregulated differentially expressed genes (DEGs) of the in‐vivo‐generated MMTs (*α*‐SMA^+^ CD68^+^ cluster) from the macrophage‐lineage scRNA‐seq were strongly associated with protumoral functions including angiogenesis and collagen fibril organization (**Figure** **4**A,B; File S1, Supporting Information), which were absent in the DEGs of transforming growth factor beta1 (TGF‐*β*1)‐generated MMTs in our previous study in vitro^[^ ^28^ ^]^(Figure S8, Supporting Information), suggesting a protumoral feature of the MMTs under cancer condition in vivo. Thus, we adoptively transferred BMDM‐derived MMTs (BMDM‐MMT) into the macrophage‐malfunctioned Nonobese diabetic/severe combined immunodeficiency (NOD/SCID) mice bearing LLC, where the BMDM‐MMT were generated in vitro by differentiating bone marrow cells into macrophages with macrophage colony‐stimulating factor (M‐CSF) followed by *α*‐SMA^+^ phenotype induction with TGF‐*β*1^[^ ^8^, ^11^, ^28^ ^]^ in vitro (Figures S9 and S10, Supporting Information). In concordance, we observed that transfer of BMDM‐MMT markedly promoted the CAF formation (*α*‐SMA^+^ cells; Figure 4C) and angiogenesis (CD31, collagen, type I (Col‐I), and basic fibroblast growth factor (bFGF); Figure 4D) in the LLC‐bearing NOD/SCID mice, resulting in a dramatic acceleration of tumor growth in vivo (Figure 4E; Figure S10, Supporting Information). The protumoral functions of MMTs were also confirmed by the macrophage‐depleted LysM‐DTR mice model, where depletion of macrophages largely reduced but adoptive transferring BMDM‐MMT significantly restored the tumor growth (Figure S11, Supporting Information); revealing a microenvironment‐dependent and disease‐specific characteristic of MMT. These results clearly demonstrated the protumoral activity of the MMT‐derived CAFs, which may represent an important therapeutic target for cancer.

### 2.5 A Smad3‐Centric Gene Network for Regulating MMT in Cancer

**Figure 5:**
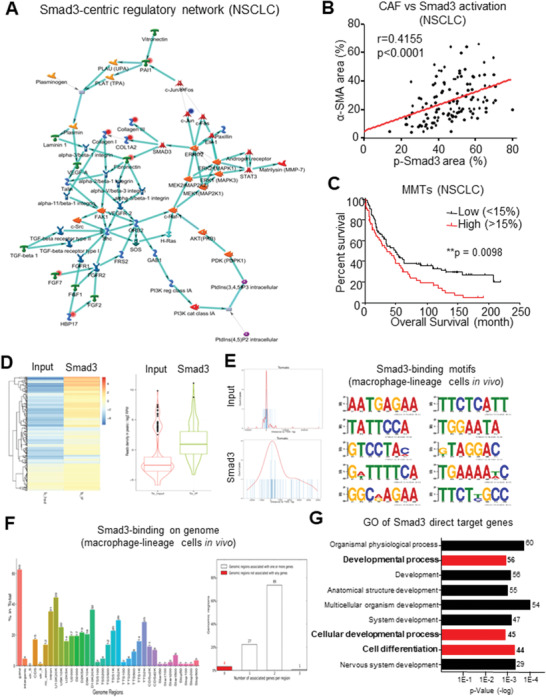
MMT is associated with Smad3‐wild‐type TME. A) A Smad3‐centric regulatory gene network is reconstructed with the upregulated DEGs of human *α*‐SMA^+^ CD68^+^ MMTs in NSCLC by MetaCore. B) Our NSCLC cohort reveals Smad3 activation level (p‐Smad3) positively correlated with CAF abundance (*α*‐SMA) (Spearman correlation, *p* < 0.0001, *n* = 121). C) Survival analysis of NSCLC cohort finds the weight of MMTs in CAFs >15% (*α*‐SMA^+^CD68^+^/*α*‐SMA^+^; *n* = 58 (MMTs/CAFs > 15%) and *n* = 103 (MMTs/CAFs < 15%)) were significantly associated with the poor overall survival of NSCLC patients (log‐rank test, *p* = 0.0098, *n* = 161). D) Heat map and box plot showing Smad3 immuno‐enrichment of genomic sequences in macrophage‐lineage cells (tdTomato^+^ cells) in LLC tumors, where cell‐type‐specific Smad3 binding preference on E) conserved sequence and F) function regions were identified by motif analysis and peak annotation. G) GO analysis (DAVID) reveals that Smad3 direct target genes were highly associated with cell developmental process and cell differentiation.

Finally, we sought to identify the key regulator of MMT in cancer by elucidating its transcriptome dynamics at single‐cell level via unbiased gene network analysis as our previous studies.^[^ ^28^, ^29^ ^]^ To this end, we extracted the upregulated genes of human *α*‐SMA^+^CD68^+^ MMTs from the public NSCLC scRNA‐seq dataset (Figure 1A; File S2, Supporting Information) and submitted to the bioinformatics platform MetaCore.^[^ ^28^ ^]^ Surprisingly, a Smad3‐centric gene network was reconstructed as the key mechanism of MMT (*p* = 7.58 × 10^−14^, *z* score = 31.55, *g* score = 112.80, **Figure** **5**A), in contrast to the Src‐centric network in kidney disease.^[^ ^28^ ^]^ Interestingly, a strong association with TGF‐*β*1‐induced myofibroblast activity was suggested by the Gene Ontology (GO) analysis from MetaCore (Figure S10, Supporting Information). Encouragingly, our cohort study detected a significant correlation between Smad3 activation and CAF abundancy in NSCLC (*p* < 0.0001, Figure 5B), where the levels of MMTs (*α*‐SMA^+^ CD68^+^ cells, *p* = 0.0098, Figure 5C) is more closely associated with the mortality of NSCLC patients compared to overall CAF abundance (*α*‐SMA^+^, *p* = 0.045; Figure S12, Supporting Information); highlighting the importance of MMT in the NSCLC progression. In concordance, bioinformatic analysis of the upregulated TGF‐*β*1/Smad3‐dependent transcriptomes of MMTs from our previous study^[^ ^28^ ^]^ found a close association with cancer in contrast to the tissue fibrosis (**Table** **1**). Moreover, we examined the importance of Smad3 in MMT‐driven CAF formation by conducting chromatin immunoprecipitation sequencing (ChIP‐seq) of the FACS‐sorted macrophage‐lineage cells from the LLC tumors of LysM‐Cre/ROSA26‐tdTomato transgenic mice (Figure 5D). We successfully obtained the binding motifs of Smad3 on the genome of macrophage‐lineage cells, where 161 direct Smad3 target genes were identified including candidates that are responsible for cell differentiation and the developmental process (Figure 5E–G; File S3, Supporting Information), highlighting the crucial regulatory role of Smad3 in the MMT‐driven CAF formation in TME.

**Table 1:** TGF‐*β*1/Smad3‐dependent transcriptome of BMDM associates with neoplasm formation. Unbiased MetaCore analysis suggests the significantly enriched disease terms (*p* < 0.05) and the number of associated TGF‐*β*1/Smad3‐dependent genes in BMDM

#	Diseases	Genes count	*p*‐value
1	Wounds and injuries	47	6.896E‐08
2	Arthritis, rheumatoid	60	9.803E‐08
3	Endocrine gland neoplasms	142	2.074E‐07
4	Pathologic processes	91	2.278E‐07
5	Rheumatic diseases	62	2.840E‐07
6	Arthritis	64	3.917E‐07
7	Immune system diseases	172	4.751E‐07
8	Rectal diseases	233	5.077E‐07
9	Intestinal neoplasms	239	5.084E‐07
10	Fibrosis	28	5.229E‐07

### 2.6 Macrophage‐Smad3 is an Effective Therapeutic Target for Eliminating MMT‐Derived CAFs

**Figure 6:**
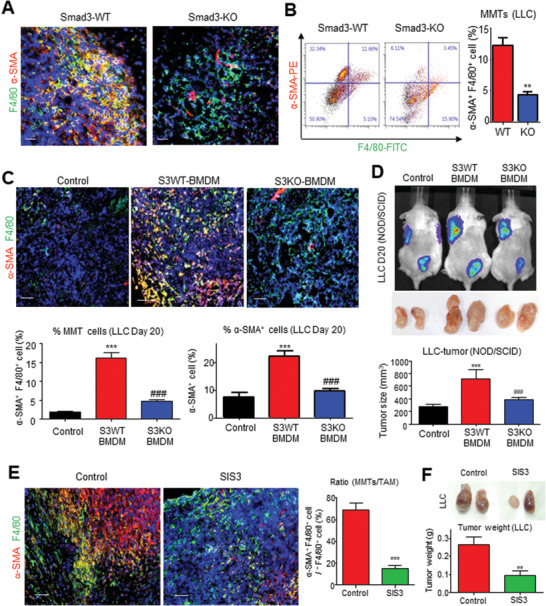
Macrophage‐specific Smad3 is a key regulator of MMT in NSCLC. Smad3 deletion significantly suppresses MMT (*α*‐SMA^+^ F4/80^+^) compared to Smad3‐WT mice: A) visualized by immunofluorescence and B) quantified by flow cytometric analysis (** *p* < 0.01 vs WT, *n* = 4, *t*‐test). C) Immunofluorescence and quantification shows that Smad3 deletion in adoptively transferred BMDM significantly suppresses the level of MMTs and *α*‐SMA^+^ CAFs in the LLC tumor of the S3KO‐BMDM group compared with S3WT‐BMDM group (***p < 0.001 vs control, ###*p* < 0.001 vs S3WT‐BMDM, one‐way ANOVA, *n* = 4). D) Live bioluminescence imaging, and tumor volume shows macrophage‐specific Smad3 blockade significantly inhibits the LLC‐luc cancer progression in S3KO‐BMDM group compared with the S3WT‐BMDM group (***p < 0.001 vs control, ###*p* < 0.001 vs S3WT‐BMDM, one‐way ANOVA, *n* = 4). Immunofluorescent shows that Smad3 inhibition by specific inhibitor SIS3 significantly suppresses E) the level of MMTs and F) tumor growth in the 5 mg kg^−1^ SIS3 treatment group compared with the solvent control group (***p < 0.001 vs control, **p < 0.01 vs control, one‐way ANOVA, *n* = 4). Scale bar, 50 µm.

To test the importance of Smad3 in macrophage‐mediated CAF formation, we examined the MMTs’ abundance in the Smad3‐knockout (Smad3‐KO) TME. Interestingly, we found a huge number of *α*‐SMA^+^ F4/80^+^ MMTs in the syngeneic LLC, which was dramatically reduced in mice lacking Smad3 (**Figure** **6**A,B), revealing TAM as a potential CAF source from the Smad3‐TME. To test the therapeutic potential of Smad3 inhibition for targeting MMT, we adoptively transferred wildtype (Smad3‐WT) or Smad3‐KO BMDM into NOD/SCID mice bearing LLC tumors. Encouragingly, the MMT‐driven CAF formation (*α*‐SMA) as well as LLC‐tumor growth in the Smad3‐WT BMDM‐received mice was significantly reduced by the macrophage‐specific silencing of Smad3 (Smad3‐KO BMDM) (Figure 6C,D), suggesting Smad3 as a potential therapeutic target for eliminating MMT‐driven CAF formation. Therefore, we applied Smad3 inhibitor SIS3 on the LLC‐bearing mice. Very encouragingly, SIS3 treatment effectively blocked MMT, CAF formation, as well as the growth of LLC tumor in vivo (Figure 6E,F).

Altogether, our findings clearly demonstrated that MMT may represent a novel therapeutic target for controlling CAF formation, which can be targeted by both macrophage‐specific and pharmaceutical inhibition of Smad3.

## 3 Discussion

Recently, we have identified a TGF‐*β*1/Smad3‐dependent mechanism in MMT in kidney diseases, where macrophages serve as a rich source for generating the fibrotic myofibroblast de novo.^[^ ^8^, ^9^, ^10^, ^24^ ^]^ Nevertheless, the potential role of MMT in the Smad3‐TME is still unexplored. Here, by scRNA‐seq analysis, we evidenced the occurrence of MMT in cancer, which significantly promoted the CAF formation and disease progression of NSCLC. TGF‐*β*1 is multifunctional in the TME, and its targeted therapy may also remove its anticancer actions that largely limit its therapeutic development. Our previous work dissected the pleiotropic effects of TGF‐*β*1 signaling, therefore, revealing the importance of Smad3 in the bone‐marrow‐derived TME.^[^ ^6^ ^]^ TGF‐*β*1/Smad3 signaling can induce CAF generation from several distinct sources, including epithelial and endothelial cells.^[^ ^30^, ^31^, ^32^, ^33^ ^]^ The pathogenic mechanism of the Smad3‐TME is still largely unknown; particularly, in CAF regulation, a better understanding may uncover new therapeutic targets for cancer. Importantly, macrophage‐specific silencing of Smad3 effectively prevented MMT, resulting in a significant reduction in CAF and tumor regression in vivo. Therapeutically, Smad3 inhibitor significantly suppresses MMT in LLC tumors, contributing to the anticancer effect of SIS3 previously reported by our group.^[^ ^6^ ^]^ In this study, we discovered a pathogenic role for MMT in cancer by promoting CAF formation directly from TAM governing by Smad3. Thus, MMT may represent a novel therapeutic target for cancer, which can be precisely blocked by targeting Smad3 in the macrophages.

Using scRNA‐seq analysis of macrophage lineage in a syngeneic mouse lung carcinoma model, LLC, we reconstructed the in vivo pseudotime course of macrophage‐lineage CAF development during tumorigenesis and further resolved their causal relationship in terms of spliced mRNA abundance (i.e., RNA velocity) via the alignment of single‐cell transcriptomes, demonstrating the de novo formation of CAFs from TAM. Indeed, TAM are highly heterogeneous, and multiple protumoral activities have been reported from their M2‐like subsets.^[^ ^29^, ^34^, ^35^ ^]^ Our study further uncovered that MMTs originate from M2 TAM with strong protumoral activities, consistent with the positive correlation between the MMT level and NSCLC mortality. We also found that MMTs accounted for >80% of the total M2 population in NSCLC biopsies and experimental LLC tumors, illustrating that M2 TAM undergo MMT to promote CAF formation.

CAFs are one of the most abundant stromal cell types in the TME. They are highly heterogeneous and multifunctional,^[^ ^30^, ^31^, ^32^ ^]^ but their origins are still largely unknown.^[^ ^33^ ^]^ The present study is the first to investigate the implication of MMT in cancer, revealing a direct mechanism of TAM for promoting CAF generation using well‐established lineage tracing strategies.^[^ ^6^, ^8^, ^9^, ^24^, ^36^ ^]^ In this study, we demonstrated the existence of MMT in cancer by identifying their co‐expression of TAM (CD68) and CAFs (*α*‐SMA) markers in the lung, kidney, and prostate cancers. The LysM‐Cre promoter is a well‐accepted macrophage specific system for lineage tracing and macrophage‐specific gene deletion;^[^ ^9^, ^24^, ^26^, ^36^, ^37^, ^38^, ^39^, ^40^, ^41^, ^42^, ^43^ ^]^ these findings are also in agreement with our data from LysM‐independent macrophage tracing and depletion. We clearly demonstrated the generation of macrophage‐lineage CAFs by fate‐mapping study using 10x scRNA‐seq, which documented the transition of macrophage‐lineage (LysM^+^) cells from *α*‐SMA^−^ F4/80^+^ (TAM) to *α*‐SMA^+^ F4/80^−^ (CAFs) in the reconstructed developmental trajectories, providing direct evidence for the *trans*differentiation of TAM to CAFs. Importantly, we further confirmed the direction of TAM lineage differentiation to CAFs by RNA velocity analysis,^[^ ^27^ ^]^ absence of LysM expression in CAFs, and reciprocal experiments on macrophage‐depleted LysM‐DTR mice and replenished by adoptive transferring of BMDM‐MMT into macrophage malfunctioned NOD/SCID mice, demonstrating TAM as a source of CAFs. Adoptive transfer of homogeneous MMTs helps us to identify the specific function of MMT in vivo. In this study, we showed that the adoptive transfer of TGF‐*β*1 stimulated BMDM markedly enhanced CAF formation and angiogenesis in the experimental TME, demonstrating plentiful of CAFs originated from macrophages during LLC‐tumor development.

Moreover, TAM are correlated with CAF abundancy in lung carcinoma and neuroblastoma.^[^ ^12^, ^13^ ^]^ Here, we found that the expression of CAF markers and protumoral effectors was largely increased in BMDM under cancer secretome stimulation in vitro, which explains the positive correlations between TAM and CAFs.^[^ ^12^, ^13^, ^44^, ^45^ ^]^ Importantly, we found that the level of MMT is significantly associated with poorer overall survival in our NSCLC cohort. This was supported by our in vivo findings that adoptive transfer of BMDM‐MMT potently promoted tumor growth of NOD/SCID mice. Interestingly, the tumor‐specific angiogenic function of MMT was revealed by GO analysis comparing DEGs from in vitro TGF‐*β*1‐generated MMTs^[^ ^28^ ^]^ and in‐vivo‐generated MMTs, which is supported largely by enhancing angiogenesis observed in LLC‐tumor‐received BMDM‐MMT transfer. In addition, the potential role of MMT‐derived CAFs in other protumor functions (e.g., adaptive immunity suppression, drug resistance, metastasis, and cancer cell stemness) is also worthy of investigation. Notably, macrophage infiltration is at a substantially lower level in the subcutaneous mouse tumor model than the highly vascularized lung tissues in human NSCLC biopsies. This can account for the observation that much higher proportions of MMTs can be found in the human NSCLC samples than in the experimental tumors. This limitation can be improved by using the orthotopic transplantation cancer model. Overall, this study helps to elucidate the underlying mechanism whereby TAM promote CAF formation, and blockade of MMT may effectively inhibit cancer progression by suppressing CAF formation during tumorigenesis.

Mechanistically, TGF‐*β*1 has a crucial role in myofibroblast generation,^[^ ^8^, ^10^, ^46^, ^47^ ^]^ but the underlying mechanism is still largely unexplored. Interestingly, by reanalyzing our scRNA‐seq data from the in‐vitro‐generated MMTs from a kidney disease study,^[^ ^28^ ^]^ we unexpectedly observed a strong association between the transcriptomes of the BMDM‐MMT and cancer. Moreover, Smad3 activation in CAFs contributes to the NSCLC progression in the NSCLC cohort studies.^[^ ^48^ ^]^ Surprisingly, unbiased gene network analysis uncovered a unique Smad3‐centric gene network as the key regulatory mechanism of MMT in NSCLC, which is different from the case of kidney disease where an Src‐centric network was identified.^[^ ^28^ ^]^ Specifically, macrophage‐specific silencing of Smad3 successfully prevented the transferred BMDM undergoing MMT in vivo, resulting in a dramatic reduction of CAF population, neo‐angiogenesis, and tumor growth.

In summary, we discovered a novel phenomenon in MMT where TAM can further *trans*differentiate into protumoral CAFs. Macrophage‐specific silencing of Smad3 effectively blocks MMT, thereby inhibiting CAF‐mediated cancer progression. These findings clearly illustrate the importance of macrophage Smad3 in CAF regulation via MMT, which may serve as a specific therapeutic target for cancer immunotherapy.

## 4 Experimental Section

### Mouse Cancer Models

All experimental procedures were approved by the Animal Experimentation Ethics Committee of the Chinese University of Hong Kong (Ref No. 20‐019‐GRF). Smad3 deficient mice (*Smad3* ^−/−^),^[^ ^49^ ^]^ macrophage‐specific tdTomato overexpression transgenic mice, NOD–SCID, and nude mice were used in this study.

For the generation of macrophage‐specific tdTomato overexpression mice, LysM‐Cre/ROSA26‐tdTomato mice were generated by crossing LysM‐Cre and ROSA26‐tdTomato mice (Jackson Laboratory, Stock Nos. 004781 and 007914). *Smad3* ^−^ *^/^* ^−^ (exon 8 deletion and exon 7 disruption) mice in C57BL/6 background were kindly provided by Dr. Chuxia Deng.^[^ ^49^ ^]^ 2 × 10^6^ LLC (C57) or A549 (nude mice) cells were injected subcutaneously (s.c.) into the right flanks of mice to induce tumors.

For macrophage‐lineage‐specific cell ablation, 150 ng per mice diphtheria toxin (Sigma, D0564) was injected intraperitoneally to conditionally deplete macrophage in LysM‐Cre Rosa26‐iDTR (LysM‐DTR) mice as described in previous studies.^[^ ^9^, ^11^, ^23^, ^50^ ^]^ Briefly, diphtheria toxin dissolved in phosphate‐buffered saline (PBS) was daily intraperitoneally injected since 3 days before LLC‐tumor inoculation until tumor collection. Clodronate and control liposomes (FormuMax Scientific Inc, F70101C‐N) were intraperitoneally injected to C57BL/6 mice 2 days before tumor inoculation (initial dose: 200 µL per mouse) and repeated every 5 days (maintenance dose: 100 µL per mouse) until tumor collection.^[^ ^25^ ^]^

### SIS3 Treatment

LLC‐tumor (s.c.) bearing Smad3^+/+^ mice were randomly divided into two groups (*n* = 8). SIS3 (S0447, Sigma) at (5 mg  kg^−1^, intraperitoneal injection (i.p.), daily) were administered until tumor collection, whereas control group was received solvent control (0.05% dimethyl sulfoxide) instead.

### 10x scRNA‐seq and Transcriptomic Analyses

tdTomato^+^ cells were fluorescence‐activated cell sorting (FACS)‐sorted from the single‐cell digestion of LLC tumor (s.c.) on LysM/tdTomato mice and submitted for cell encapsulation and library construction by a chromium controller with 5′ expression kit (10x genomics). The library was sequenced by an illumina NovaSeq 6000 platform (PE151bp, 660M raw read). The sequence data were converted into cloupe format by Cell Ranger v3.0.2, and DEGs of MMTs were generated by Loupe Cell Browser software. Upregulated DEGs of MMTs were submitted to Database for Annotation, Visualization, and Integrated Discovery Bioinformatics resources (DAVID v6.8) for Gene Ontology biological processes enrichment analysis. Developmental trajectories were reconstructed by Monocle 2 package. RNA velocity analysis was performed by scVelo to infer future states of individual cells using the spliced and unspliced information. The aligned bam file generated by Cell Ranger was recounted with the Velocyto counting pipeline velocyto.py in python. The complete code and notebooks of Velocyto and scVelo used is available at http://velocyto.org. and https://scvelo.readthedocs.io

To elucidate the regulatory mechanism of MMTs in human NSCLC, upregulated DEGs of *α*‐SMA^+^ CD68^+^ MMTs were extracted from the NSCLC dataset by Loupe Cell Browser software and submitted to MetaCore analytical suite for unbiased Kyoto Encyclopedia of Genes and Genomes (KEGG) pathway and network analysis. Gene networks were reconstructed from the unique DEGs of MMTs based on published evidence, revealing significant interaction between DEGs and crucial regulators, including transcription factors and receptors. All selections were filtered by a *p*‐value of <0.05. The publicly available NSCLC datasets (V(D)J + 5' Gene Expression ‐ Human (v1 Chemistry), Cell Ranger 2.2.0, NSCLC tumor ‐ 5’ gene expression) can be downloaded from 10x genomics. via the following URL: https://www.10xgenomics.com/resources/datasets/nsclc‐tumor‐5‐gene‐expression‐1‐standard‐2‐2‐0

### Flow Cytometric Analysis

Tissues isolated from tumor‐bearing mice and NSCLC patients were mechanistically dissociated, digested by Liberase (Roche), filtered by 40 µm nylon mesh, and fixed with Intracellular (IC) fixation buffer (eBioscience) according to manufacturer's protocol to prepare a single‐cell suspension. MMTs were stained with fluorescent antibodies against *α*‐SMA, CD206, CD68, and F4/80 (**Table** **2**) overnight at 4 °C. Flow cytometric data were acquired on LSRFortessa (Becton Dickinson) and analyzed in the Cytobank platform (cytobank.org) for quantitative analysis.

**Table 2:** List of antibodies used. Details of antibodies used in western blot, immunofluorescent staining, ChIP assay, and immuno‐histochemistry

Target	Concentration	Catalog number	Manufacturer	Application
*α*‐SMA	1:1000	m0851	Dako	Western blotting
GAPDH	1:10 000	mab374	Millipore	
VEGF	1:500	sc152	Santa Cruz	
F4/80	1:100	Allophycocyanin (APC): 17‐4801‐82 Phycoerythrin (PE): 12‐4801‐82 Fluorescein isothiocyanate (FITC): 11‐4801‐85	eBioscience	Immunofluorescent staining
*α*‐SMA	1:300	F3777	Sigma	
CD31	1:100	102514	Biolegend	
hCD68	1:100	F7135	Dako	
mCd68	1:100	137012	Biolegend	
CD206	1:300	AF488: 141710 APC: 141708	Biolegend	
p‐Smad3	1:200	600‐401‐919	Rockland	
bFGF	1:100	sc74412	Santa Cruz	
Anti‐GFP	1:20	338008	Biolegend	
Col‐1	1:100	1310‐01	SouthernBiotech	
p‐Smad3	1:100	sc‐517575	Santa Cruz	IHC
*α*‐SMA	1:100	M0851	DAKO	
CD68	1:100	M0814	DAKO	
Smad3	1:50	9523S	Cell Signaling Technology	ChIP

### Cell Culture

The LLC (CRL‐1642, ATCC) cells with stable luciferase expression (LLC‐luc), 3T3 (ATCC CRL‐1658), A549 (ATCC CCL‐185) were cultured in Dulbecco's Modified Eagle Medium/Nutrient Mixture F‐12 (DMEM/F12) and DMEM, respectively, with 10% heat‐inactivated fetal bovine serum (FBS) (Gibco), 100 U mL^−1^ penicillin G, and 100 mg mL^−1^ streptomycin. LLC cancer cells conditioned medium (LLC‐CM) was collected by overnight by the incubation of LLC cells with serum‐free DMEM/F12, followed by filtration with 0.2 µm nylon membrane.

BMDM were prepared following the well‐established protocol.^[^ ^8^, ^9^, ^11^, ^28^ ^]^ In brief, single‐cell suspension of bone marrow cells was prepared from tibia, femur, and ilium bone. The bone marrow cells were differentiated 7 days in DMEM/F12 with 10% heat‐inactivated FBS and 50 ng mL^−1^ recombinant mouse macrophage colony‐stimulating factor (Gibco). The purity of BMDM culture was validated by flow cytometry as the first scRNA‐seq study,^[^ ^28^ ^]^ where >95% of the cells were expressing F4/80. BMDM were further polarized to M2 by 24 h IL‐4 (20 ng mL^−1^, Gibco) stimulation.^[^ ^26^ ^]^

### ChIP‐Seq Analysis

1.5 × 10^6^ fluorescence‐activated cell‐sorting isolated tdTomato^+^ cells (day 15, LLC tumor, LysM‐Cre ROSA26‐tdTomato mice) were processed by SimpleChIP Enzymatic Chromatin IP Kit (Cell Signaling Technology) according to the manufacturer's instruction.^[^ ^6^, ^51^ ^]^ Smad3 immunoenriched DNAs (input and Smad3‐IP) were sequenced by an illumina HiSeq platform (PE150bp, 40M raw read), mapped against GRCm38 (mm10) *mus musculus* genome using bowtie2, peak called using model‐based analysis for ChIP‐seq (MACS) with default parameters. Top 100 binding regions were annotated by Genomic Regions Enrichment of Annotations Tool (GREAT) and submitted to DAVID‐GO enrichment analysis with a *p*‐value cutoff of <0.001.

### Adoptive Transfer Studies

To investigate the specific role of MMT, *Smad3^+/+^* BMDM were further induced by TGF‐*β*1 (5 ng mL^−1^) for 5 days to generate BMDM‐MMT with *α*‐SMA^+^ expression and spindle‐like morphology as shown in Figure S9 (Supporting Information) for an adoptive transfer study.^[^ ^8^, ^28^ ^]^ ^52^ In brief, BMDM‐MMT were mixed with LLC cells in a 1:1 ratio (2 × 10^6^ cells per mouse) for subcutaneous inoculation to induce LLC tumor on macrophage malfunction NOD–SCID mice, and diphtheria‐toxin‐driven macrophage depleted LysM‐DTR mice. To determine the TAM‐specific role of Smad3 in MMT‐mediated tumor progression, PBS, *Smad3* ^+/+^, and *Smad3* ^−/−^ BMDM were mixed with LLC cells in 1:1 ratio (2 × 10^6^ cells per mouse), respectively, to induce subcutaneous tumor for the three groups: control, Smad3‐WT BMDM, and Smad3‐KO BMDM. Tumor volume was measured every 2 days with a Vernier caliper and calculated using the equation: Volume (mm^3^) = 0.5 (long × square of short diameter). Before tumor collection, bioluminescence imaging of LLC‐luc tumors was conducted in the IVIS Spectrum system (Caliper, Xenogen).

### Quantitative Real‐Time PCR

TRI reagent (Molecular Research Center), reverse transcription system (Promega), and SYBR Green Supermix (Bio‐Rad) were used for RNA extraction, complementary DNA (cDNA) synthesis, and real‐time polymerase chain reaction (PCR), respectively, according to manufacturer's protocol.^[^ ^53^, ^54^ ^]^ *α*‐SMA, VEGF, CD31, F4/80, LysM, and glyceraldehyde 3‐phosphate dehydrogenase (GAPDH) primers used in this study are listed in **Table** **3**. Gene expression levels normalized with GAPDH of three experiments were expressed as mean± standard error of the mean (s.e.m.).

**Table 3:** List of primers used. Details of primers used in real‐time PCR

	Forward primer (5′ to 3′)	Reverse primer (5′ to 3′)
GAPDH	GCATGGCCTTCCGTGTTC	GATGTCATCATACTTGGCAGGTTT
VEGF	CCGGTTTAAATCCTGGAGCG	TTTAACTCAAGCTGCCTCGC
CD31	TGCTCTCGAAGCCCAGTATT	TGTGAATGTTGCTGGGTCAT
*α*‐SMA	GTGCTATGTCGCTCTGGACTTTGA	ATGAAAGATGGCTGGAAGAGGGTC
F4/80	TCTGGGGAGCTTACGATGGA	GAATCCCGCAATGATGGCAC
Cd68	CTTCCCACAGGCAGCACAG	AATGATGAGAGGCAGCAAGAGG
LysM	ATTGCAGTGCTCTGCTGC	GTGAGAAAGAGACCGAATG

### Western Blotting

The tissues or cells were lysed by radioimmunoprecipitation assay (RIPA) Lysis Buffer System (Santa Cruz). The protein concentration was measured by DC protein assay (Bio‐Rad), and 40 µg of protein was electrophoresed in 10% sodium dodecyl sulphate (SDS)–polyacrylamide gel (Bio‐Rad) and blotted to nitrocellulose membrane (Pall Corporation). The membrane was incubated with *α*‐SMA, VEGF, and GAPDH antibodies overnight 4 °C. The bound antibody was detected by dylight‐800 conjugated secondary antibody (Rockland) with Odyssey infrared imaging system (LI‐COR). The expression level of the target protein was quantified by ImageJ of three repeated experiments and normalized to the internal control (GAPDH).

### Immunofluorescence

Optimal cutting temperature (OCT)‐compound‐embedded tumor tissues (5 µm thickness) were used for immuno‐histochemical staining. After OCT removal and blocking, sections were incubated with primary antibody overnight at 4 °C. For nonconjugated primary antibody, sections were incubated with Alexa‐Fluor 488/546‐conjugated secondary antibody (1:1000, Invitrogen) in staining buffer (Invitrogen) for 2 h at room temperature. Nuclei were stained with Hoechst 33342, then mounted with PermaFluor medium (Thermo Fisher Scientific). Images were captured by a Zeiss fluorescence microscope and analyzed by ZEN image analysis software.

### Opal Multiplex Immuno‐Histochemistry

Formalin‐fixed paraffin‐embedded tumor microarray (TMA) was deparaffinized and rehydrated, followed by endogenous horseradish peroxidase (HRP) blocking (3% hydrogen peroxide solution, 30 min) and heat‐induced epitope retrieval in citrate buffer (95 °C, 5 min). After incubation with primary antibody overnight (4 °C), the fluorescence was developed using OPAL 4‐color fIHC kit (Perkin Elmer, MA) according to the manufacturer's protocol. TMA was imaged on the Mantra quantitative pathology workstation (Perkin Elmer, MA) and analyzed by inForm image analysis software (Perkin Elmer, MA). The formalin‐fixed paraffin‐embedded blocks of primary NSCLC samples in Prince of Wales Hospital, the Chinese University of Hong Kong (CUHK), were used for the study, under the approved protocols (Reference No. 2019.368) by CUHK Clinical Research Ethics Committee, and written informed consent was obtained from patients.

### Statistical Analysis

Student's *t*‐test or analysis of variance with Bonferroni post test was used to perform statistical analysis of the differences in mRNA expression levels, tumor weight and growth, vessel count, protein levels, and percentage or ratio of target cells in Prism program (Prism 5.0 GraphPad Software, San Diego, CA). Data were presented as mean ± s.e.m. . Pearson and Spearman correlation analyses and Comparison of Survival Curves with Log‐rank Test were used to analyze NSCLC cohort data; *p* < 0.05 was considered statistically significant.

## Conflict of Interest

The authors declare no conflict of interest.

## Supporting information

Supporting Information

Supplemental File 1

Supplemental File 2

Supplemental File 3

## Data Availability

The data that support the findings of this study are available from the corresponding author upon reasonable request.
