# Supplementary material for: Smad3 Promotes Cancer‐Associated Fibroblasts Generation via Macrophage–Myofibroblast Transition
Source: Adv Sci (Weinh). 2021 Nov 17;9(1):2101235. doi: 10.1002/advs.202101235 (PMC8728853; doi:10.1002/advs.202101235)
Supplement: Supplementary file 1 — Supporting Information [file ADVS-9-2101235-s003.pdf]

ADVANCED SCIENCE
Open Access

Supporting Information

for *Adv. Sci.*, DOI: 10.1002/advs.202101235

Smad3 Promotes Cancer-Associated Fibroblasts Generation via Macrophage-Myofibroblast Transition

Philip Chiu-Tsun Tang, Jeff Yat-Fai Chung, Vivian Wei-wen Xue, Jun Xiao, Xiao-Ming Meng, Xiao-Ru Huang, Shuang Zhou, Alex Siu-Wing Chan, Anna Chi Man Tsang, Alfred Sze-Lok Cheng, Tin-Lap Lee, Kam-Tong Leung, Eric W-F Lam, Ka-Fai To, Patrick Ming-Kuen Tang, and Hui-Yao Lan**

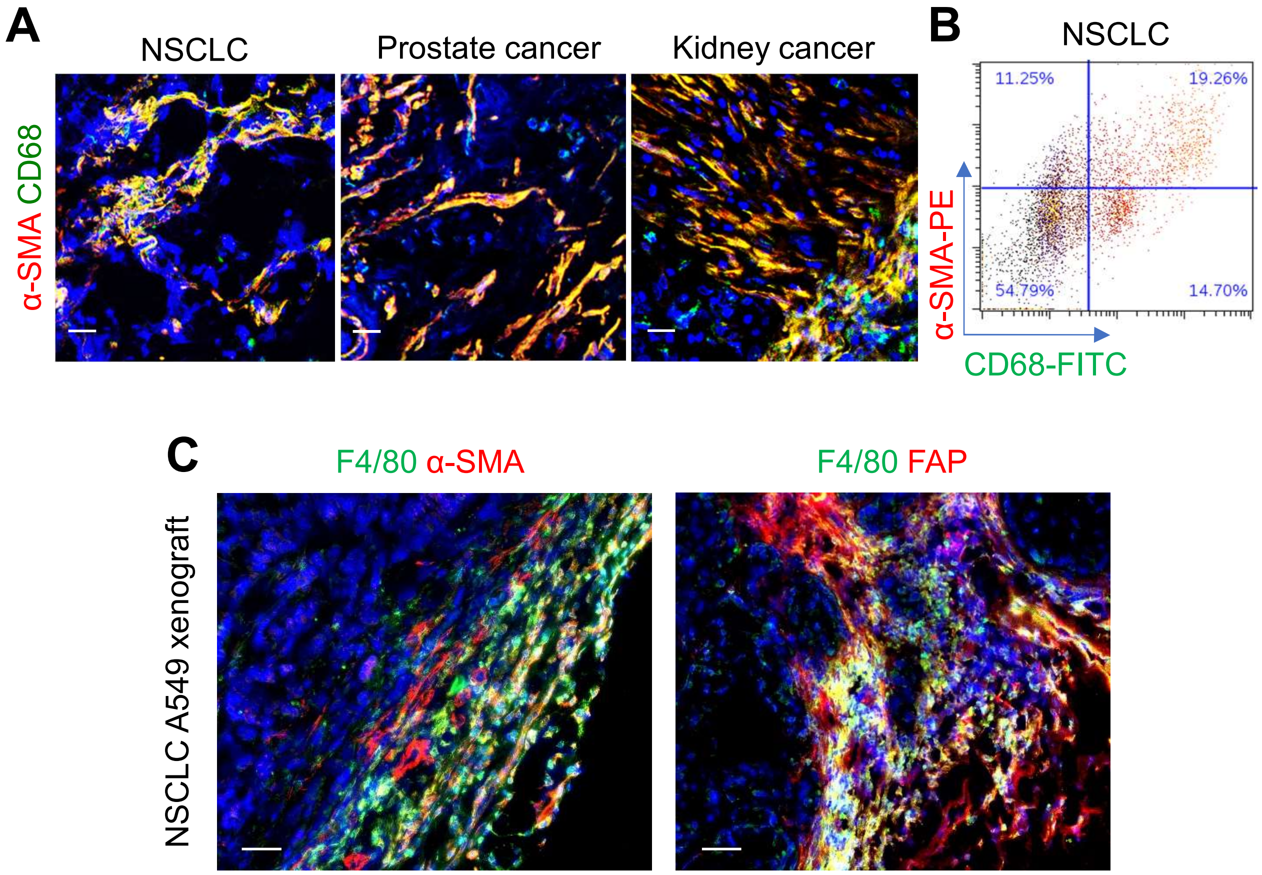

Supplementary Figure S1. MMT exists in human TME. CAF marker (α-SMA) expressing TAM (CD68) in NSCLC, prostate and kidney cancer specimens were detected by (**A**) confocal imaging and (**B**) flow cytometry. **(C)** CAF marker (α-SMA or FAP) expressing TAM (F4/80) were detected in human NSCLC A549 xenograft bearing nude mice. Scale bar, (**A, C**) 50 μm

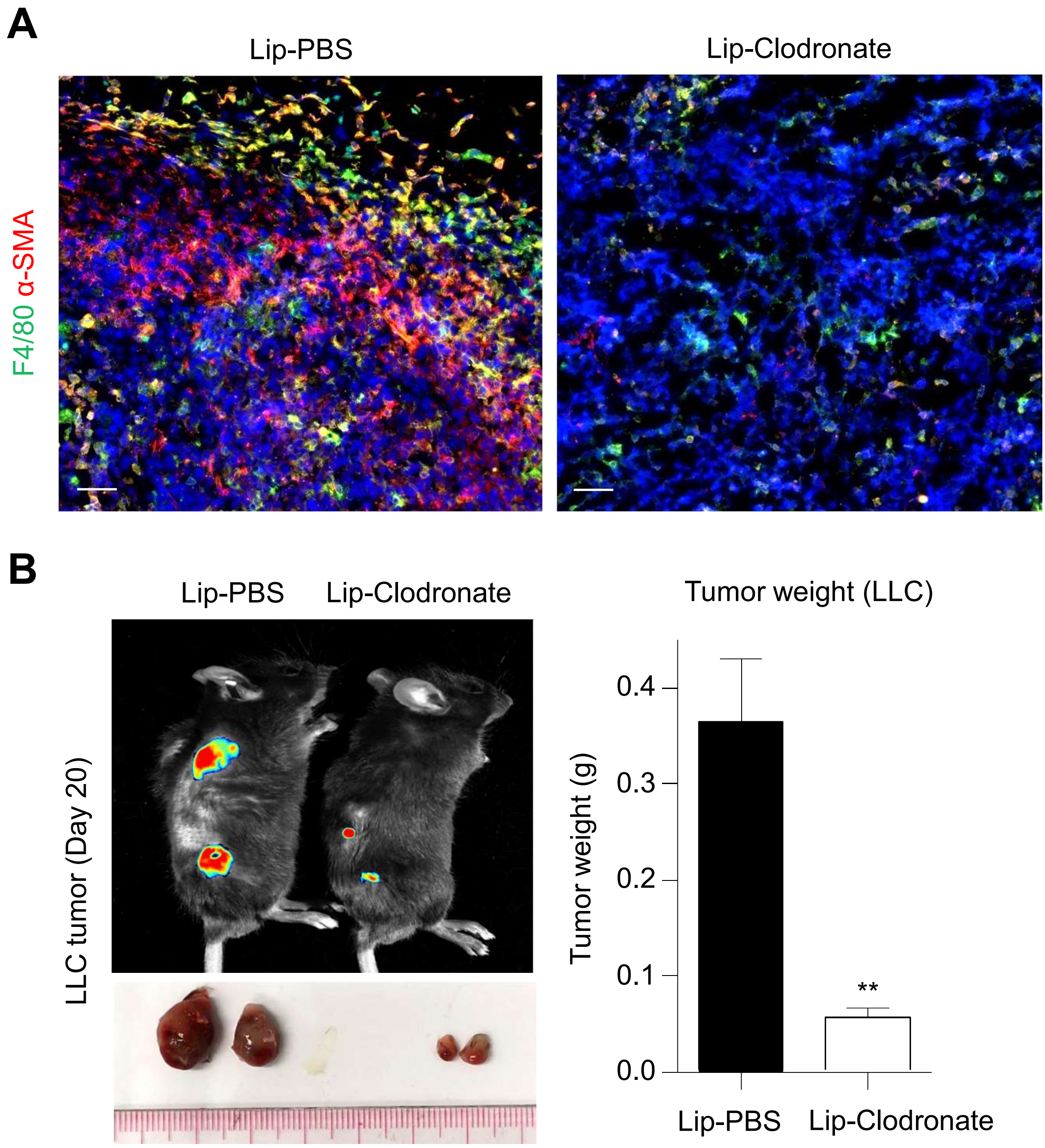

Supplementary Figure S2. Clodronate liposome mediated macrophage depletion largely reduced MMT *in vivo*. (**A**) Clodronate liposomes (Lip-Clodronate) effectively depleted TAM (F4/80) and reduced MMTs (α-SMA F4/80) in the LLC-tumor *in vivo*, (**B**) associated with a markedly reduction of tumor growth compared to the control mice treated with PBS liposomes (Lip-PBS; **p<0.01 vs Lip-PBS, n=4, t-test). Scale bar, (**A**) 50 µm.

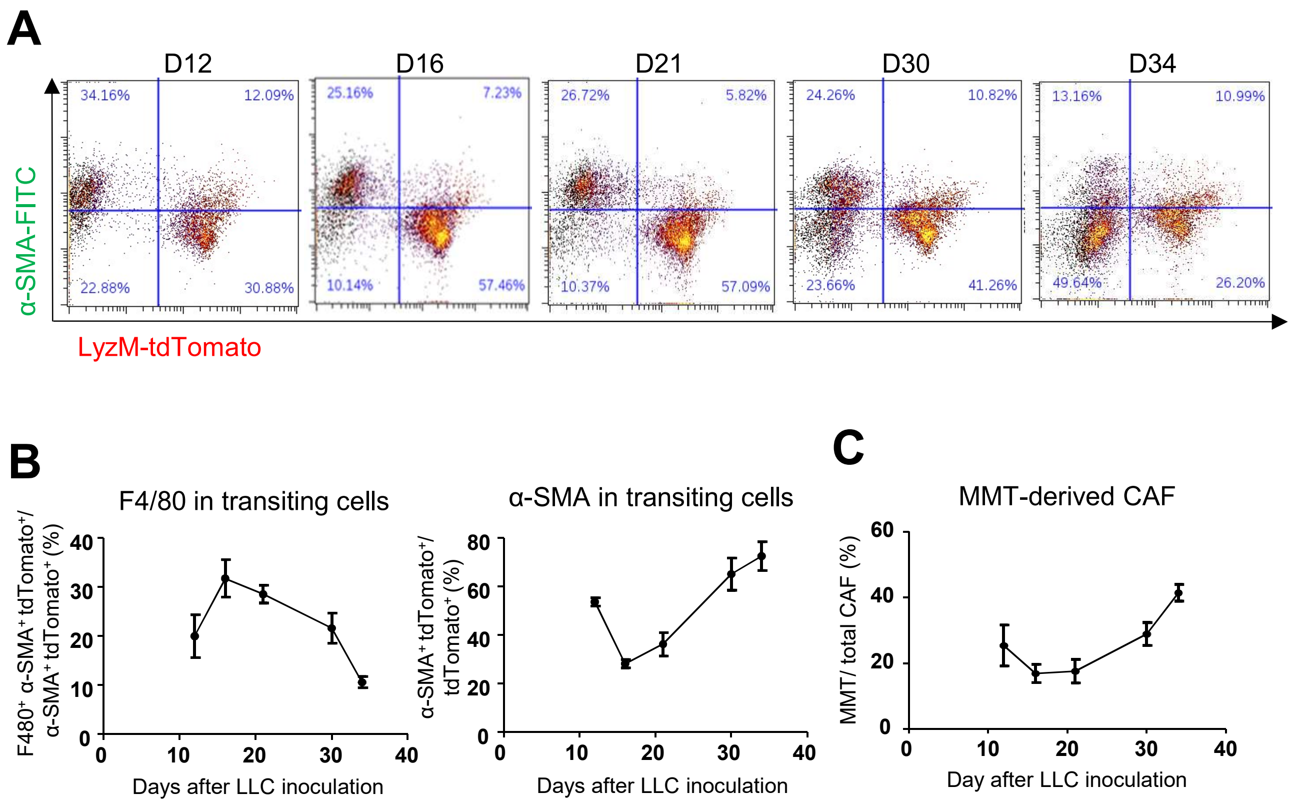

Supplementary Figure S3. Increment of macrophage-lineage CAFs during tumorigenesis. (**A**) Flow cytometry detected macrophage-lineage derived CAFs (α-SMA$^+$ tdTomato$^+$) during LLC tumor progression. Interestingly, their quantifications showed that (**B**) the predominate expression of macrophage marker (F4/80, left panel) in tdTomato$^+$ cells was switched to be CAF marker (α-SMA, right panel) at the late stage of the tumorigenesis *in vivo*, accounting for (**C**) ~40% of total CAFs on Day 34 (n=3-4).

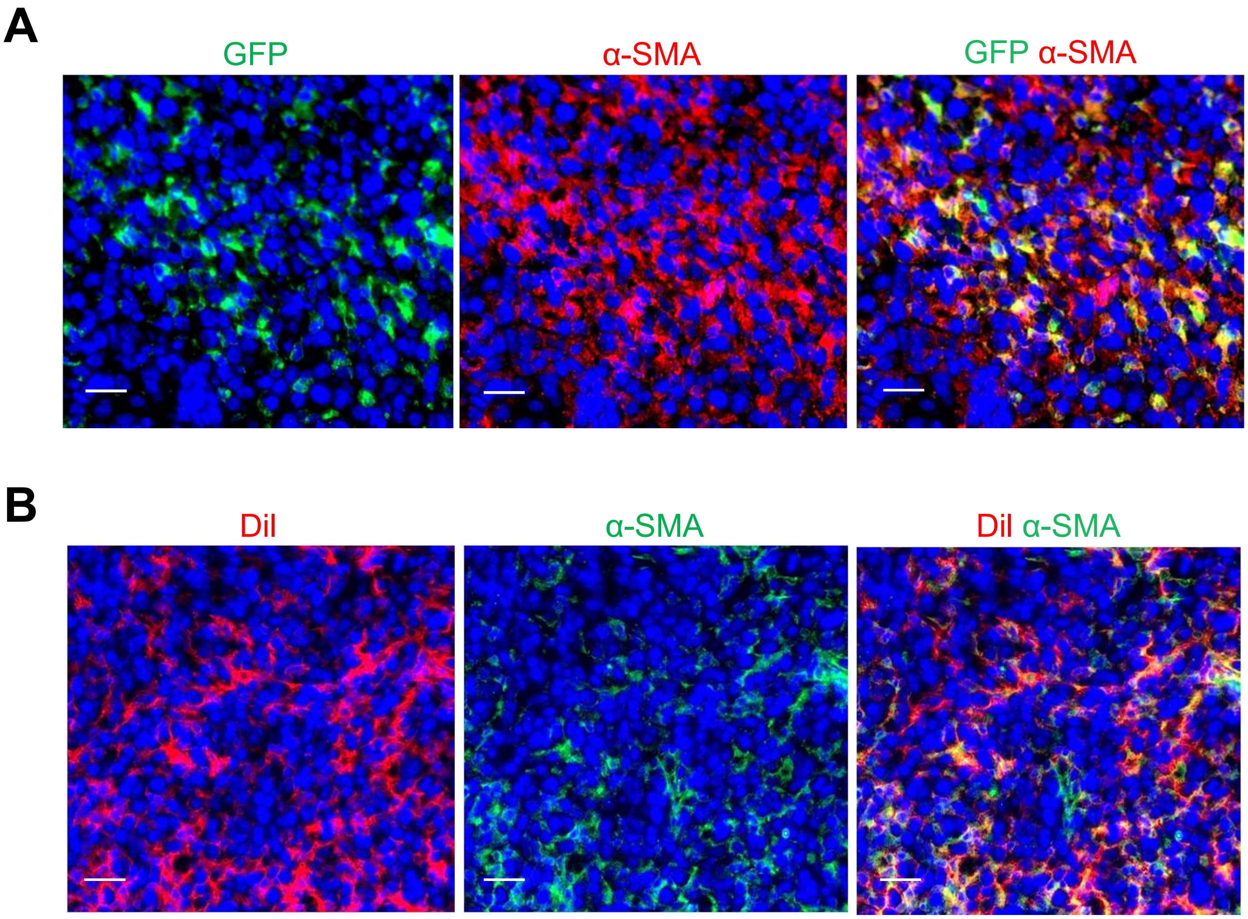

Supplementary Figure S4. The existence of BMDM-derived CAFs *in vivo*. (**A**) GFP expressing or (**B**) Dil tracker dye stained BMDMs were adoptively transferred into the LLC-bearing mice, their derived α-SMA^{+} CAFs were detected in tumors on day 25 by immunofluorescence. Scale bar, (**A, B**) 50 μm.

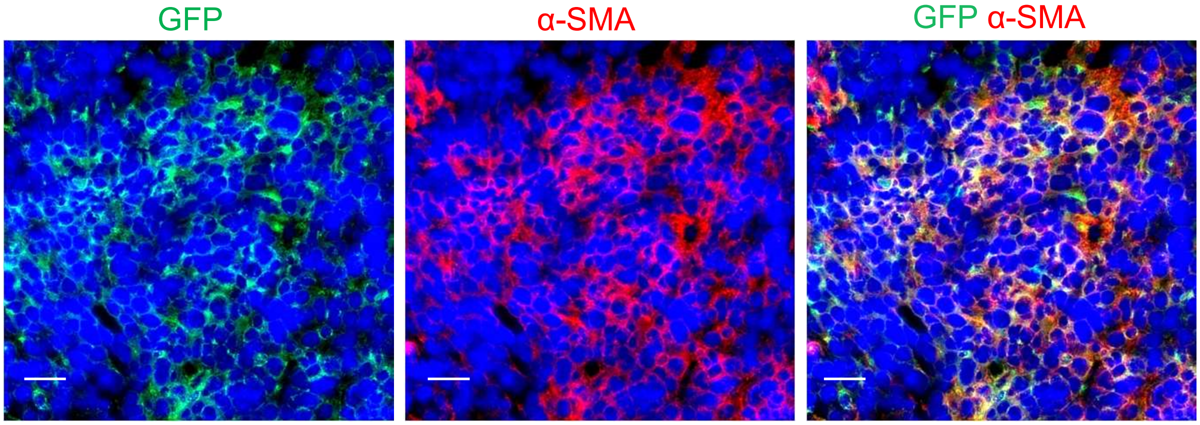

Supplementary Figure S5. M2-derived CAF exists in the LLC-tumor *in vivo.* GFP expressing BMDM-generated M2 cells (polarized by IL-4 *in vitro*) were adoptively transferred into the LLC-bearing mice, their derived α-SMA^{+} CAFs were found in the tumors on day 25 by immunofluorescence. Scale bar, 50 μm.

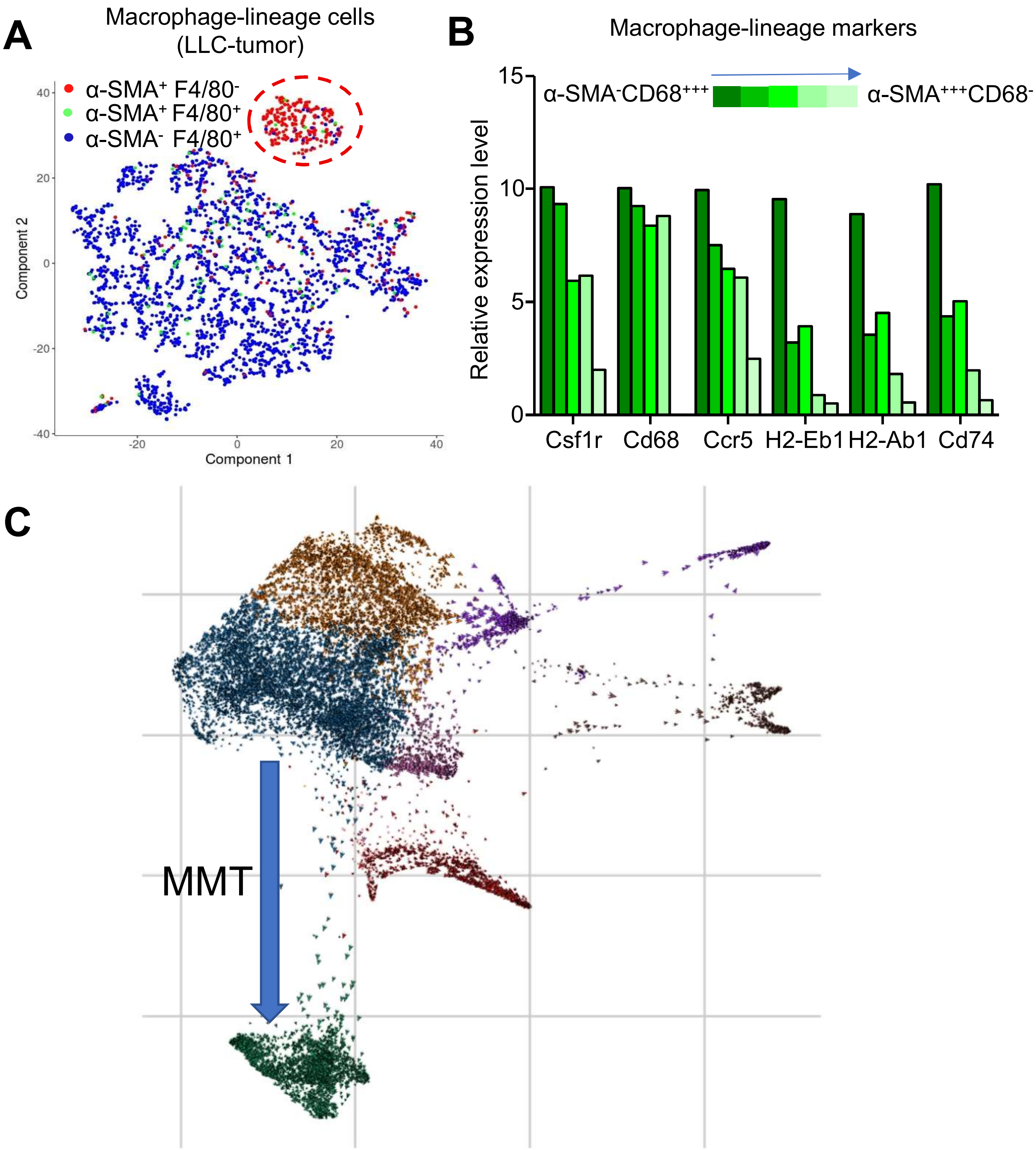

Supplementary Figure S6. RNA velocity analysis recapitulates dynamics of MMT in the LLC-tumor *in vivo*. (**A**) A unique CAF cluster (α-SMA^{+}) was found from the macrophage-specific 10x scRNA-seq of the LysM-driven tdTomato^{+} cells sorted from LLC-tumor. (**B**) Interestingly, we observed a progressive loss of macrophage lineage markers (Csf1r, Cd68, Ccr5, MHC II molecules (H2-Eb1, H2-Ab1, Cd74) in the sorted LysM-driven tdTomato^{+} cells, from the strongest TAM phenotype (α-SMA^{-}CD68^{+++}) to CAF phenotype (α-SMA^{+++}CD68^{-}) in LLC-tumor on Day 16. (**C**) RNA velocity analysis recapitulated the dynamics of macrophage-lineage cells, revealing the *de novo* generation of CAFs (green cluster) from the TAMs (brown and blue clusters) in the LLC-tumor at transcriptome level with single cell resolution.

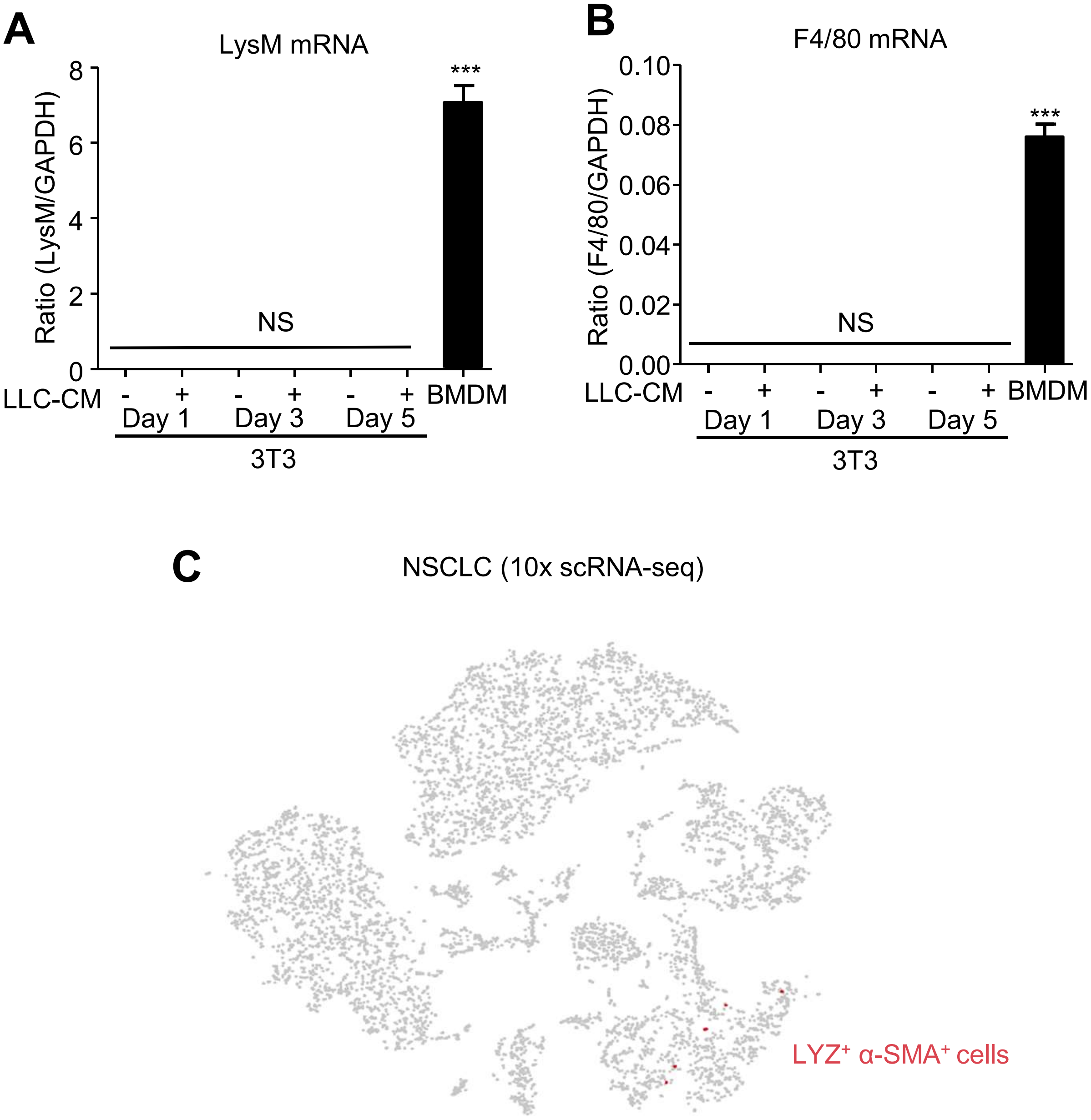

Supplementary Figure S7. Expression of LysM is undetectably low in fibroblast. The mouse fibroblasts (NIH/3T3, ATCC CRL-1658) and BMDMs were stimulated with LLC-CM for 1, 3, and 5 days. By real-time PCR analysis, we found that the expression level of macrophage markers **(A)** LysM and **(B)** F4/80) were undetectable in the LLC-CM stimulated mouse NIH/3T3 fibroblasts (ATCC CRL-1658) compared to BMDM (NS $p>0.05$ vs control groups without LLC-CM stimulation, ***$p<0.001$ vs 3T3 fibroblast groups, one-way ANOVA, n=3). **(C)** In addition, expression of LYZ (human gene homologue of the mouse LysM) was extremely low in the α-SMA$^+$ CAF cluster of the human NSCLC dataset used in Figure 1A.

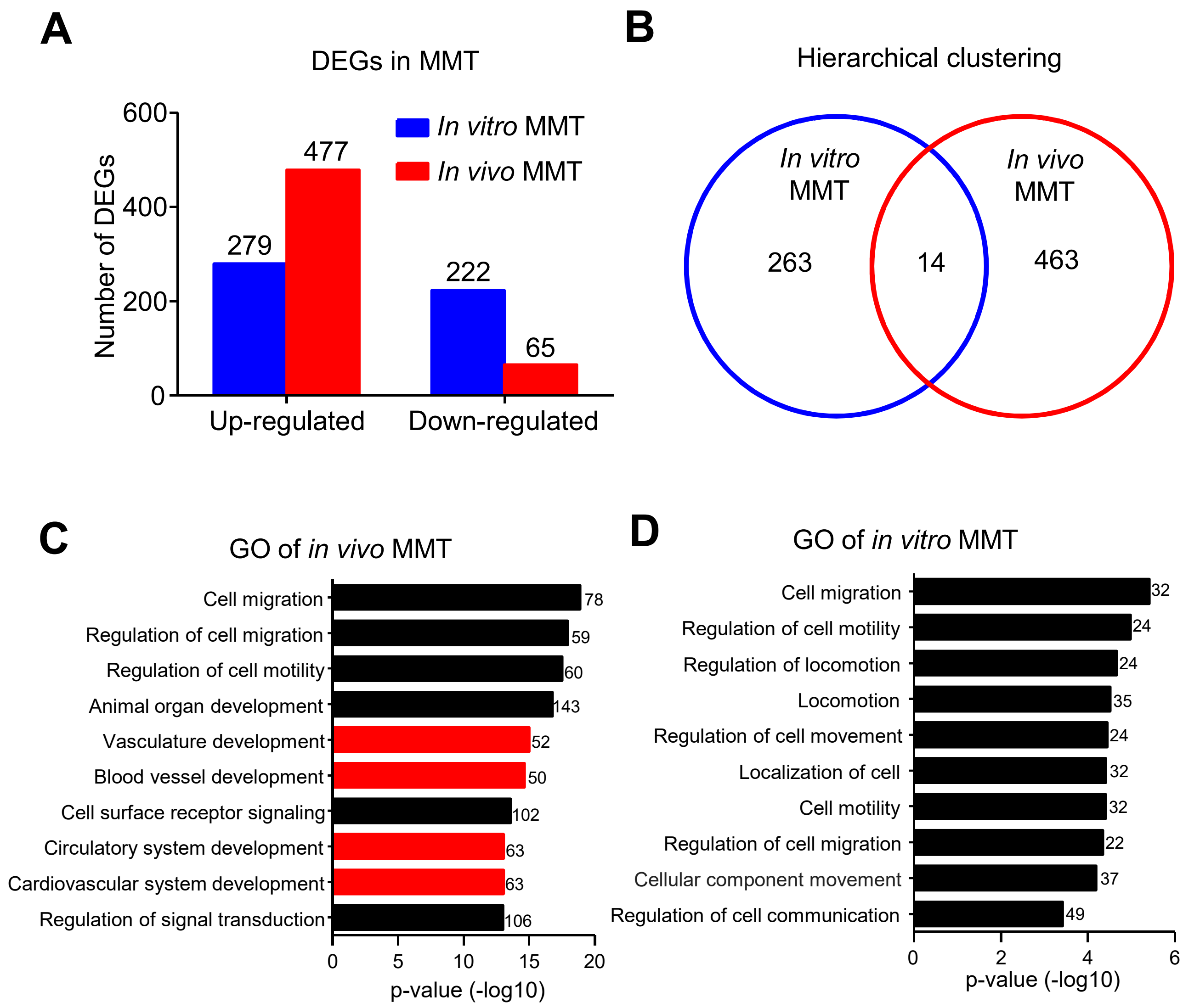

Supplementary Figure S8. Transcriptome analysis revealed the protumoral phenotypes that are specifically showed in the *in vivo* generated MMTs. (A) The up- and down-regulated DEGs of the MMTs compared to their controls, BMDMs without TGF-β1 stimulation *in vitro*[28] and α-SMA^{-ve} F4/80^{+} TAMs (Figure 4A) *in vivo*, were extracted from their scRNA-seq datasets. **(B)** The numbers of common and MMT-specific DEGs were shown as a hierarchical clustering diagram. GO analysis of the up-regulated DEGs found specifically in the **(C)** *in vivo* and **(D)** *in vitro* generated MMTs, where results associated with protumoral functions were highlighted in red.

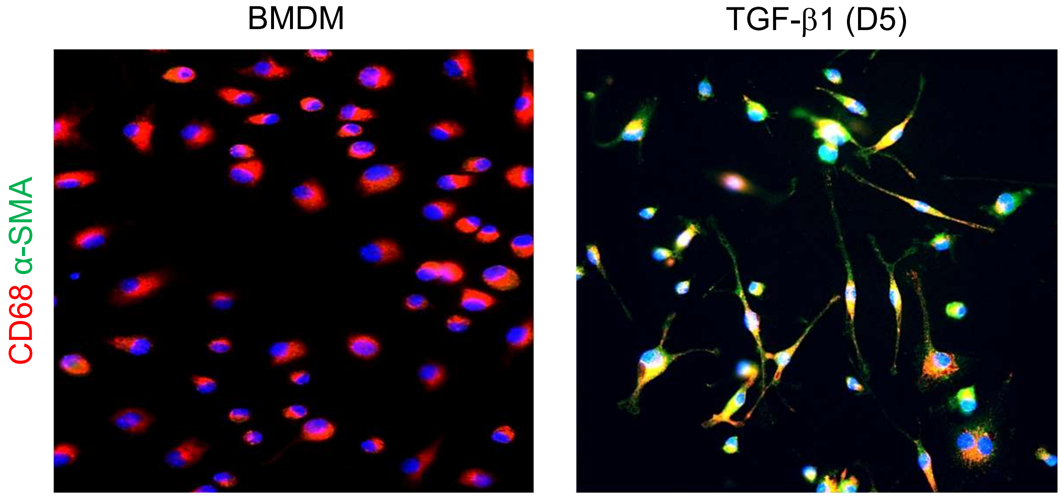

Supplementary Figure S9. Protein expression of α-SMA in the *in vitro* generated MMTs. Immunofluorescence detected a strong co-expression of both CAF (α-SMA) and macrophage (CD68) markers in the MMTs generated from BMDMs under 5 days of TGF-β1 stimulation *in vitro*.

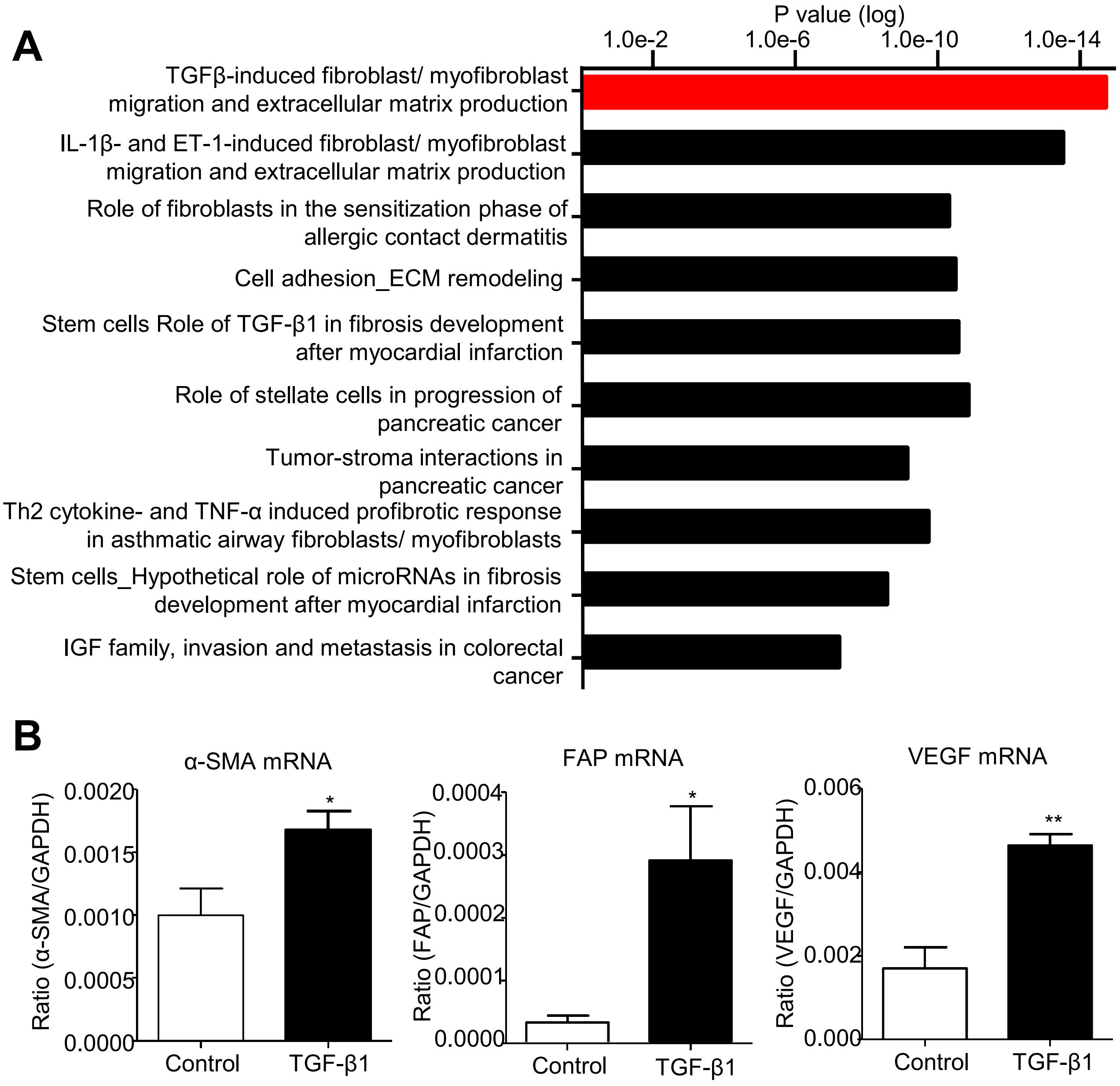

Supplementary Figure S10. TGF-β1 signaling in TAMs is important for inducing CAF phenotypes. (A) Unbiased bioinformatic platform MetaCore (Clarivate Analytics) highlighted the importance of TGFβ-induced myofibroblast activity in the MMTs of NSCLC by submitting the up-regulated DEGs of α-SMA^{+}CD68^{+} cells from the dataset of Figure 1A to a KEGG pathway analysis. **(B)** Furthermore, our results showed that TGF-β1 (5 ng/ml) markedly induced the expression of myofibroblast markers (α-SMA and FAP) and effector (VEGF) in the BMDMs on day 5 *in vitro* (**p<0.01, * p<0.05 vs control, t-test, n=3).

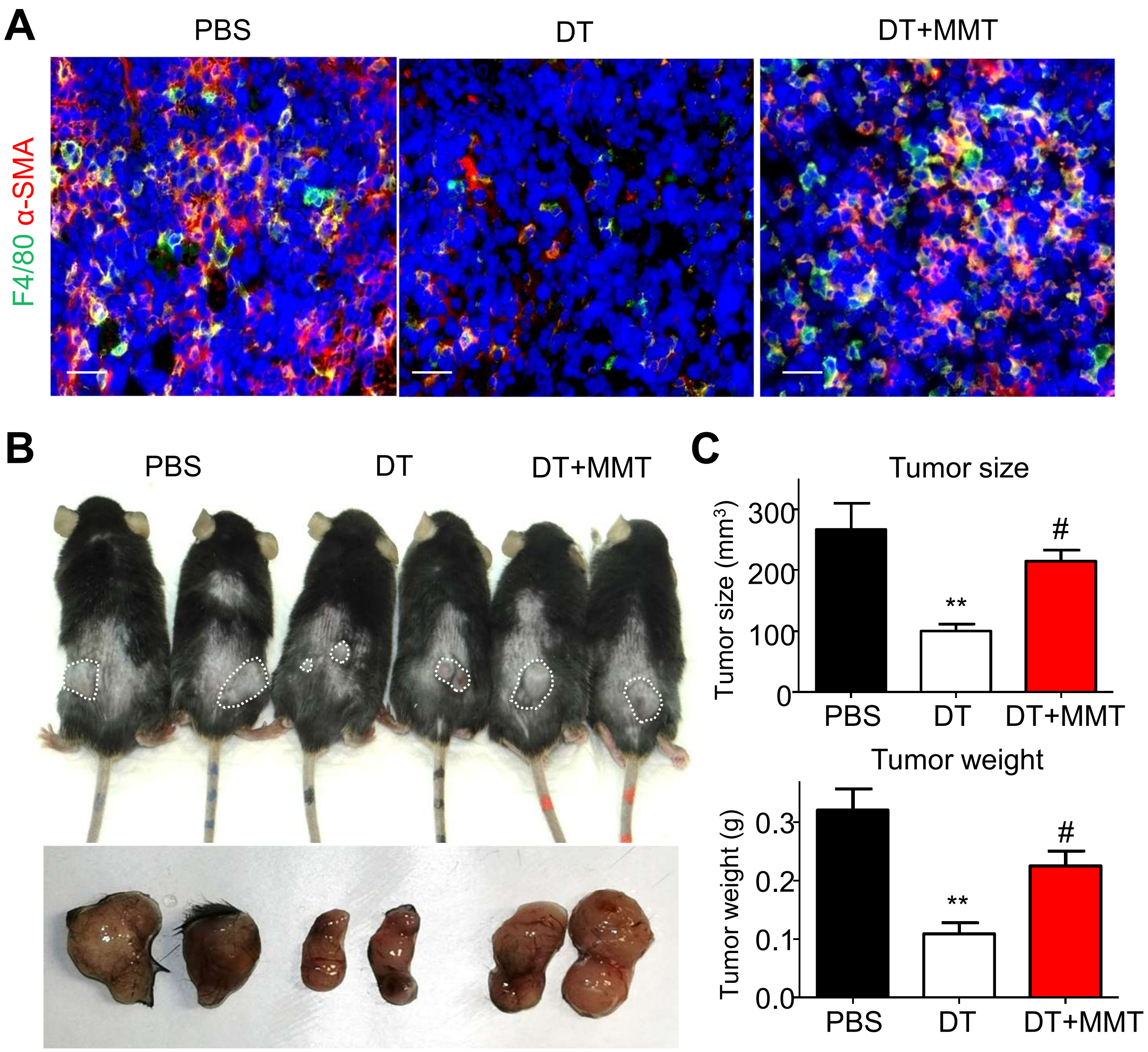

Supplementary Figure S11. Protumoral effect of MMTs in the macrophage-depleted mice. Diphtheria toxin mediated macrophage depletion largely suppressed **(A)** MMT as well as **(B, C)** tumor growth of the LLC-bearing LysM-DTR mice (DT) compared to their control group (PBS), which was effectively restored in the depleted mice by adoptive transferring the *in vitro* generated MMTs (DT+MMT) (**p<0.01 vs PBS control, #p<0.05 vs DT, one-way ANOVA, n=3-4). Scale bar, (**A**) 50 μm.

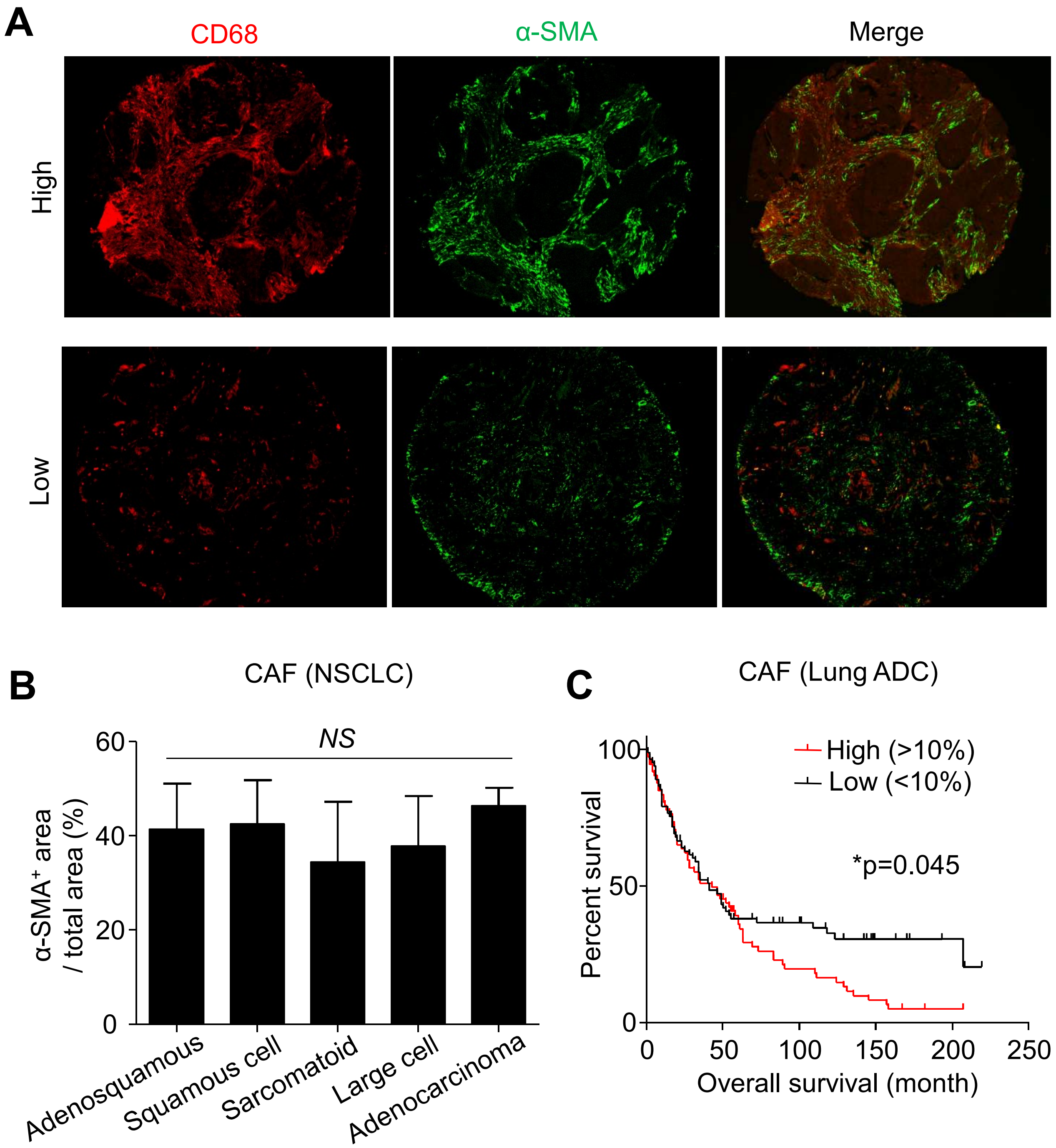

Supplementary Figure S12. Level of overall α-SMA$^+$ CAFs showed a weak association with the mortality of lung adenocarcinoma. (A) Representative images of specimens with high and low levels of MMT (α-SMA$^+$ CD68$^+$) in the NSCLC cohort of Figures 2 and 5. **(B)** No significant difference of the overall α-SMA$^+$ CAFs between subtypes was detected in the same NSCLC cohort (NS, one-way ANOVA, n=222), **(C)** where high level of overall CAFs (α-SMA$^+$ area > 10%) was weakly associated with a poorer survival of the patients with lung adenocarcinoma (*p=0.045, Log-rank test, n=161).
